# Repetitive stress-related injury of the proximal metacarpus in a seven-year old Thoroughbred racehorse with emphasis on diagnostic analgesia of the proximopalmar metacarpus

**DOI:** 10.1186/2046-0481-67-26

**Published:** 2014-12-01

**Authors:** Felim MacEoin, Paul Robinson

**Affiliations:** Department of Veterinary Clinical Services, The Hong Kong Jockey Club, Sha Tin Racecourse, New Territories, Hong Kong, China SAR

## Abstract

This report describes the diagnosis and management of repetitive stress-related injury (RSRI) of the proximal metacarpus in a seven-year old Thoroughbred racehorse. Intra-articular analgesia of the middle carpal joint (MCJ) as well as perineural analgesia of the lateral palmar nerve (LPN) abolished lameness and a diagnosis of RSRI of the proximopalmar metacarpus was made after nuclear scintigraphic examination. Given the response to intra-articular analgesia, the authors undertook a cadaver study in order to better describe the relationship between the medial and lateral palmar pouches of carpo-metacarpal joint (CMCJ), the LPN and the deep branch of the lateral palmar nerve (DBLPaN).

## Background

Repetitive stress-related injury (RSRI) of the proximopalmar aspect of the third metacarpal bone (McIII) in Thoroughbred racehorses is a potentially catastrophic cause of forelimb lameness [1] and this injury is not always radiographically evident [1–3]. Diagnosis often relies on advanced imaging techniques such as nuclear scintigraphy [1], magnetic resonance imaging (MRI) [4] or computed tomography (CT) [5]. A thorough lameness investigation that includes diagnostic analgesia is first required to localise the site of pain causing lameness to the proximopalmar metacarpal region [6, 7].

A prompt and accurate diagnosis of RSRI of the proximopalmar metacarpus is important to prevent the possibility of a catastrophic fracture [3, 6]. Innervation of the palmar aspect of the proximal metacarpus in the horse is complex [1, 7, 8] and lameness due to injury in this location can result in protracted periods of rest and rehabilitation [4, 9]. In addition, adjunctive therapies instigated on the basis of a misdiagnosis can prove costly to owners and result in a poor outcome. Advanced imaging modalities such as MRI and nuclear scintigraphy have enabled a far greater understanding of the relationship between diagnostic analgesia and the vast array of pathology that can result in equine lameness [3, 4, 9].

## Case presentation

### Introduction

A seven-year-old Thoroughbred gelding that was in full preparation for racing was presented for investigation of acute-onset, grade 3/5 [10] right forelimb lameness on two separate occasions following galloping approximately 2 months apart. Detailed clinical examination failed to reveal any obvious site of lameness. On both occasions, perineural analgesia of the lateral and medial palmar and palmar metacarpal nerves at the level of the distal second and fourth metacarpal bones (low 4-point nerve block) was performed using a total volume of 8 ml of mepivicaine hydrochloride 2% (Intra-epicaine)^2^. This failed to alleviate the lameness although a positive response to MCJ analgesia using 6 ml of mepivicaine via a dorsal approach was a consistent finding. At the time of the second occurrence of lameness, analgesia of the lateral palmar nerve (LPN) was performed via a medial approach using 2 ml of mepivicaine, which abolished the lameness. This was undertaken 24 hours after the positive response to intrasynovial analgesia of the MCJ. The medial approach to the LPN was chosen because it is less likely to cause inadvertent penetration of local synovial structures than other methods [11].

### First episode of lameness

Following a positive response to analgesia of the MCJ, a radiographic examination of the right carpus and proximal metacarpus was performed and repeated after 10 days of stall confinement. No significant abnormalities were identified on either radiographic study and after this period of stall confinement the horse showed no lameness at a walk or trot. Given the lack of clinical and radiographic abnormalities, the horse resumed training and raced 4 weeks later.

### Second episode of lameness

Two weeks after this race, the horse became acutely lame (3/5) in the right forelimb after galloping. A low 4-point nerve block using mepivicaine did not improve the lameness although intra-articular analgesia of the MCJ abolished the lameness. Radiography of the carpus was repeated and no significant bony abnormality was detected. The following day, perineural analgesia of the lateral palmar nerve (LPN) via a medial approach on the axial aspect of the accessory carpal bone (ACB) [12] was performed using 2 ml of mepivicaine that also abolished the lameness. The efficacy of the nerve block was confirmed by lack of skin sensation on the lateral heel bulb.

### Diagnostic imaging

**Figure 1:**
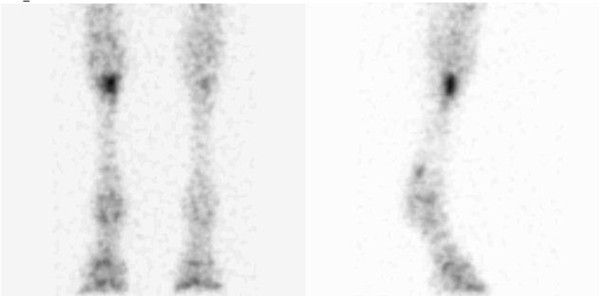
**Increased IRU in the proximopalmar right metacarpus**.

Serial radiographic studies did not reveal any pathology of the carpus or proximal metacarpus during both episodes of lameness although a primary bone injury was still considered likely. For this reason, nuclear scintigraphy was elected to further elucidate the cause of lameness. The horse underwent nuclear scintigraphic (MiE Scintron VI)^3^ examination 7 days after perineural analgesia of the LPN. Objective image analysis was performed using MiE supplied motion correction software (Paralyzer)^3^ to compare count ratios after drawing a rectangular region of interest (ROI) on the lame and contralateral limb. The ROI was drawn on the proximal metacarpus so as to include the area of increased radiopharmaceutical uptake (IRU). There was a 150% increase in count ratio between the limbs, which is considered abnormal [3, 13] (Figure 1) and a diagnosis of RSRI of the proximopalmar cortex of McIII was made.

### Management

Following this diagnosis, the horse was confined to a stall for six weeks after which the lameness had fully resolved. At this time, a radiographic examination of the proximal metacarpus did not reveal any abnormalities and the horse resumed training. He raced a total of four times after the diagnosis of an RSRI was made. The first race was three months after the nuclear scintigraphic examination and he was unplaced. Over the following four months he won and placed in 2 of the remaining 3 starts. A soft tissue injury of the deep digital flexor tendon in the distal metacarpus of the right forelimb was sustained in the fourth race and he was retired for this reason.

### Cadaver study

#### Introduction

The authors performed a cadaver study to clarify the response of the current case to the diagnostic analgesic procedures undertaken. It has been suggested that inadvertent desensitisation of adjacent structures following intrasynovial analgesia can occur, leading to an inaccurate diagnosis of the site causing lameness. This is thought to occur through diffusion of local anaesthetic across a synovial membrane or by subcutaneous leakage from the injection site [14]. The relationships between the palmar aspect of the carpo-metacarpal joint (CMCJ), lateral palmar nerve (LPN) and the deep branch of the lateral palmar nerve (DBLPaN) were examined in detail.

## Materials and methods

**Figure 2:**
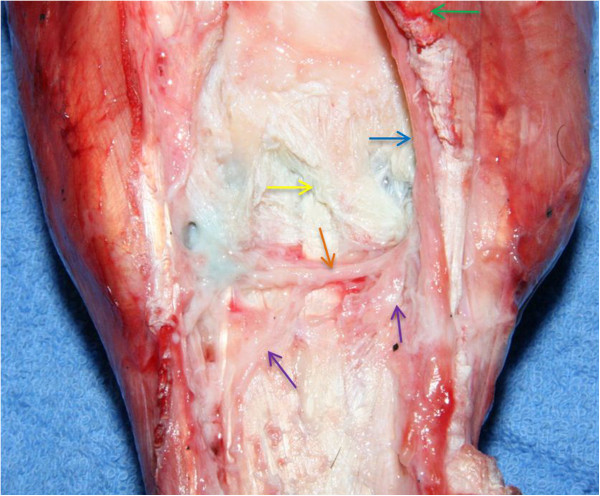
**Right forelimb showing the accessory carpal bone** **(green arrow),** **origin of the inferior check ligament after removal** **(yellow arrow),** **lateral palmar nerve** **(blue arrow)** **lying axial to the accessoriometacarpal ligament**, **deep branch of the lateral palmar nerve** **(orange arrow),** **lateral and medial palmar metacarpal nerves** **(purple arrows).**

Six pairs of cadaver forelimbs were obtained from Thoroughbred racehorses (average weight 510 kg; range 469-576 kg) euthanised for reasons unrelated to lameness. Each limb was dissected to expose the palmar pouches of the CMCJ, the LPN, the DBLPaN and the medial and lateral palmar metacarpal nerves (Figure 2). Methylene blue (6 ml) was injected into the MCJ via a dorsal approach to facilitate identification of the palmar pouches of the CMCJ. Removal of the superficial digital flexor tendon and a portion of the deep digital flexor tendon (DDFT) from the carpal canal allowed visualisation of the inferior check ligament (ICL). This was transected at the level of its attachment to the DDFT and reflected proximally to expose the deep volar arch, the CMCJ pouches and regional nerves.

**Figure 3:**
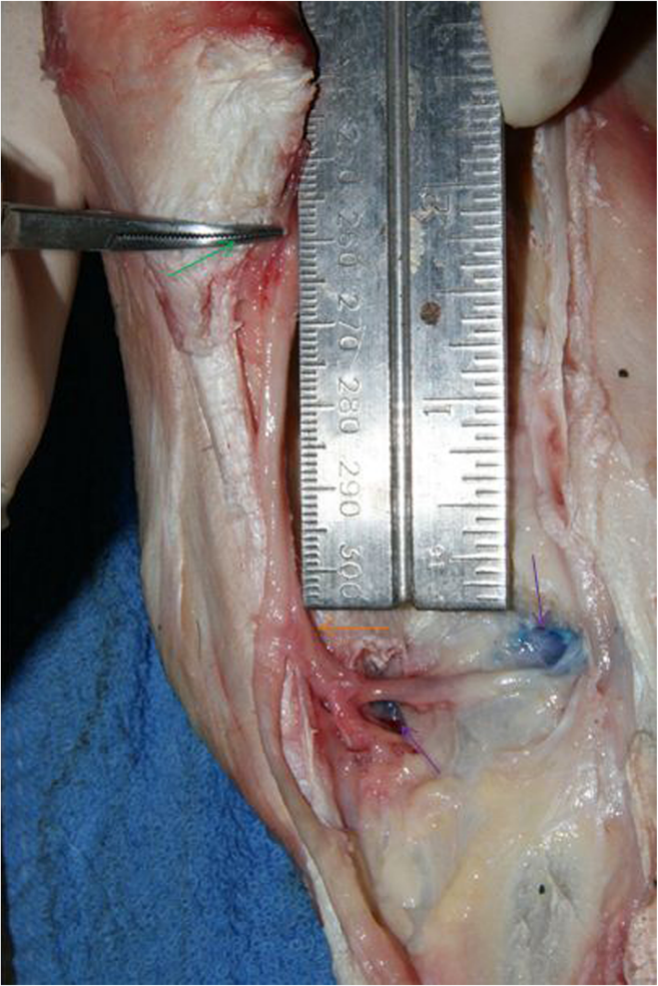
**Left forelimb showing distance from distal ACB (green arrow) to DBLPaN (orange arrow) and from the DBLPaN to lateral and medial pouches of CMCJ (purple arrows).**

Using a standard metric ruler from the distal margin of the ACB, the distance at which the DBLPaN arose from the LPN and the distance to the deep volar arch [15] were measured and recorded (Figure 3). In addition, the distance between the DBLPaN and the medial and lateral palmar pouches of the CMCJ was also recorded. All measurements for each anatomical variable were combined and the mean and range were calculated (Table 1).

**Table 1:** **Summary of neuroanatomical data relating to the proximopalmar metacarpus**

	Weight (kg)	ACB to branch	ACB to volar arch	Nerve to lateral CMC jt.	Nerve to Medial CMC Jt.
**Horse no. 1**	**512**				
Left		20	55	0	0
Right		20	55	0	0
**Horse no. 2**	**576**				
Left		35	50	5	0
Right		30	60	0	0
**Horse no. 3**	**502**				
Left		40	55	0	0
Right		43	55	5	2
**Horse no. 4**	**469**				
Left		12	45	0	0
Right		25	55	0	0
**Horse no. 5**	**491**				
Left		35	55	0	0
Right		15	55	0	0
**Horse no. 6**	**509**				
Left		20	55	0	0
Right		40	55	0	0
Mean		27.92	54.17	0.84 mm	0 mm
Range		12 mm - 43 mm	45 mm - 60 mm	0 mm - 5 mm	0-2 mm

**Figure 4:**
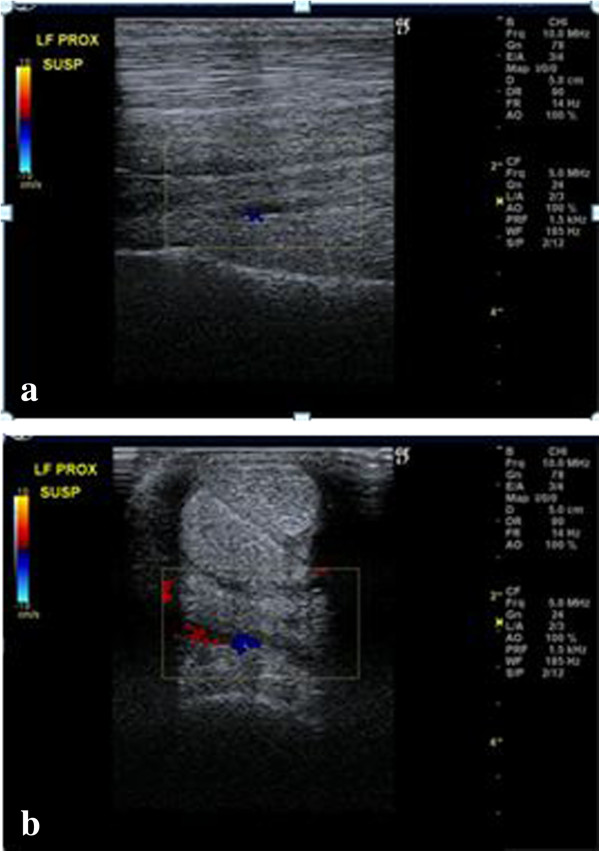
**Weight-bearing a) longitudinal and b) transverse images of the proximopalmar metacarpus highlighting the deep volar arch using Doppler.** Proximal is to the left on the longitudinal image and lateral is to the left on the transverse image.

**Figure 5:**
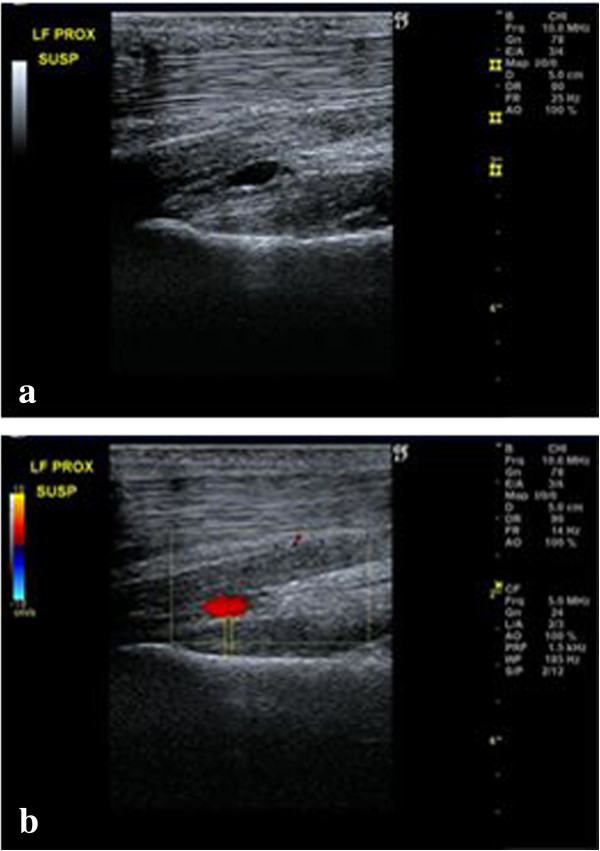
**Non** **weight-bearing longitudinal images showing the deep volar arch a) without and b) with Doppler.**

Ultrasonographic^4^ examination of the forelimbs of a live horse was then performed to identify the deep volar arch which was facilitated using Doppler with the limb in a weight bearing (Figure 4) and non-weight bearing stance (Figure 5).

### Institutional review board

This study was performed on cadaver specimens of horses euthanized for unrelated reasons and as such approval from an ethics committee was not sought.

## Results

**Figure 6:**
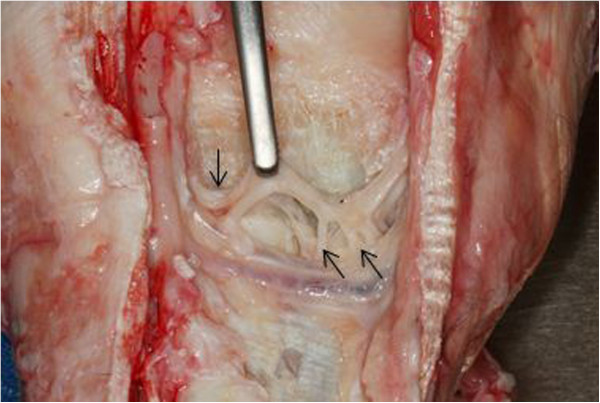
**Right forelimb showing several smaller branches of the DBLPaN (arrows).** Lateral is to the right of the image.

The DBLPaN arose from the LPN on average 27.9 mm (range 12 - 43 mm) distal to the base of the ACB before coursing horizontally in a lateral to medial direction (Figure 3). In all specimens, several smaller nerves arose from the DBLPaN and entered the proximal suspensory ligament (PSL) between the larger lateral and medial palmar metacarpal nerves (Figure 6).

**Figure 7:**
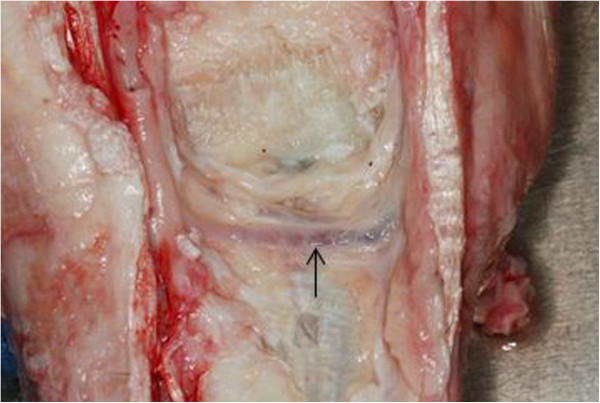
**Right forelimb showing the deep volar arch** **(black arrow)** **immediately distal to the DBLPaN.** Lateral is to the right of the image.

The average distance between the distal margin of the ACB and the deep volar arch was 54 mm (range 45 - 60 mm). The horizontal portion of the DBLPaN was parallel and immediately proximal to the deep volar arch in every limb (see Figure 7).

The DBrLPN was located within 5 mm of the lateral (average 0.83 mm; range 0 - 5 mm) palmar pouch and was in direct contact with the medial pouch of the CMCJ in all specimens (see Figure 3).

## Discussion

### Case report

This report describes the diagnosis and management of a Thoroughbred racehorse with an RSRI of the proximopalmar cortex of McIII. The diagnosis was made using nuclear scintigraphy after localising the region of lameness through diagnostic analgesia. The authors refer to this condition as an RSRI rather than a “stress fracture” because of the lack of a distinct fracture on any of the radiographic studies performed [2]. Lameness in this horse was moderate (3/5) and acute in onset and was abolished by MCJ analgesia on both occasions. Perineural analgesia of the LPN was performed at the time of the second episode of lameness and yielded a positive result. Proximal diffusion of local anaesthetic following perineural analgesia of the LPN has been described and the potential for desensitisation of the distal radius and palmar carpus should always be considered [11].

It is unclear whether both episodes of lameness had a similar aetiology. However, given the similar clinical examination findings, a positive response to MCJ analgesia and lack of obvious radiographic abnormalities on both occasions, we think it is reasonable to speculate that they were. An ultrasonographic examination of the proximopalmar metacarpus was considered. However, given the nature of the lameness, together with the lack of evidence of inflammation of the proximal suspensory region, we chose to undertake a nuclear scintigraphic examination, which we felt allowed us to make an accurate diagnosis.

In our racing population, horses that present with similar lameness that responds to analgesia of this region are frequently diagnosed with a bone injury. This may be apparent immediately on radiographs as an incomplete fracture or an area of intense resorption. Alternatively, in some cases no abnormalities can be detected for 10 to14 days, after which time these features can become radiographically evident in association with early bone resorption [2]. If the lameness has fully resolved and follow-up radiographic examination is unremarkable after this lag period, a gentle return to exercise is typically recommended, with advice that the horse is monitored closely. In this case, these recommendations were followed and the horse responded favourably to a short period of rest and raced approximately 40 days after the initial episode of lameness.

Repetitive stress-related injuries of bone can be successfully managed conservatively with rest followed by a controlled return to exercise [16].

In this case, a second episode of moderate, acute-onset lameness prompted a nuclear scintigraphic examination after diagnostic analgesia localised the site of lameness to the proximopalmar metacarpal region. Having reached a diagnosis of RSRI of the proximopalmar McIII, six weeks of stall confinement was advised after which time the horse was free of lameness. Serial radiographic examinations did not reveal any evidence of bone pathology. The 150% increase in count-ratio of the ROI between the affected and non-affected limbs is considered a mild IRU and as such, radiographic evidence of a fracture would not be expected [3].

### Cadaver study

The authors also undertook a cadaver study in order to clarify the anatomical relationship between the location of the DBLPaN and the palmar pouches of the CMCJ. The MCJ and CMCJ always communicate [7, 17] and this is also illustrated with the methylene blue being visualized in the palmar CMCJ pouches after injection of the MCJ in these twelve cadaver specimens. Our findings demonstrate that the DBLPaN courses horizontally and parallel to the deep volar arch from lateral to medial on the palmar aspect of the PSL after branching from the LPN distal to the ACB. This is in contrast to illustrations that represent the DBLPaN as an inverted ‘V’ [12] at this location. The DBLPaN lies on average within 1 mm of the lateral pouch of the palmar CMCJ and is in direct contact with the medial pouch of the palmar CMCJ, which is in agreement with previous cadaver studies [8, 17]. The authors used 6 ml of 0.5% methylene blue solution to identify the palmar pouches of the CMCJ for three reasons: to reflect an acceptable volume of local anaesthetic used by clinicians during intra-articular analgesia; to easily identify the palmar carpal joint pouches; and to provide a volume that would not distort the anatomical relationship of the palmar pouches of the CMCJ and the DBLPaN. The level at which the DBLPaN arises from the LPN distal to the ACB has been reported previously [3, 12, 18]. The current study confirmed that the DBLPaN arises distal to the ACB but at varying distances (see Table 1). This makes specific perineural analgesia of the DBLPaN potentially problematic in the live horse. This variation in distance should be taken into account in cases where surgical transection of the DBLPaN is performed for treatment of PSL desmitis [18]. Multiple, small branches of the DBLPaN were visible in all specimens entering the palmar PSL (Figure 6). These probably represent sensory innervation of the PSL and McIII and further histological study into their significance is warranted.

### Diagnostic analgesia

Subcutaneous leakage and diffusion across a synovial membrane have been proposed as two possible causes of perineural desensitisation when intrasynovial analgesia is performed [14, 19, 20]. In a recent study, intrathecal administration of local anaesthetic and contrast solution into the digital flexor tendon sheath resulted in desensitisation of the heel bulbs in more than one third of the limbs [14]. This illustrates that perineural desensitisation can occur and should be considered following intrasynovial diagnostic analgesia. In the present case, a dorsal approach to the MCJ was used discounting the theory of subcutaneous leakage causing perineural analgesia of the LPN or DBrLPN. The authors therefore believe that the most likely explanation for the improvement in lameness was by diffusion of local anaesthetic across the palmar CMCJ capsule resulting in perineural analgesia of the DBrLPN. An alternative theory is that local anaesthetic diffused through the joint surface and subchondral bone, or that there was direct communication between the site of RSRI and the CMCJ. However, for this to be the case, some disruption of normal radiographic anatomy would have been expected.

### Ultrasonography

Locating the deep volar arch via ultrasonography allows the position of the horizontal portion of the DBLPaN to be accurately located. The DBLPaN was identified immediately proximal and parallel to the deep volar arch in all cadaver specimens (Figure 7). Additionally, the deep volar arch can be used as a location marker when measuring dorsal to palmar thickness of the PSL when injury of this structure is suspected (Figure 4).

## Conclusion

Clinicians should consider the possibility of an RSRI of the proximopalmar aspect of the third metacarpal bone in a lame horse that responds favorably to intra-articular analgesia of the MCJ and in the absence of radiographic findings. Diffusion of local anaesthetic across the palmar CMCJ capsule to desensitise the DBLPaN is the most likely explanation for this response when a dorsal intrasynovial approach is used. This cadaver study showed that the DBLPaN lies in close apposition to the palmar pouches of the CMCJ. Perineural analgesia of the LPN should be performed to further elucidate the site of lameness and if a soft tissue injury is suspected to involve the PSL, ultrasonography should be carried out.

The location of the DBLPaN can be accurately located based on its position relative to the deep volar arch which is easily identified ultrasonographically. Nuclear scintigraphy is a valuable diagnostic modality when an RSRI of the metacarpus is suspected. This is especially true when radiographic findings consistent with a stress fracture are absent on successive examinations.
